# Oncothermia and Integrative Medicine—A Novel Paradigm for Infratentorial Meningioma Management: A Case Report With One-Year Follow-Up

**DOI:** 10.7759/cureus.77005

**Published:** 2025-01-06

**Authors:** Pradeep MK Nair, Ayyappan Palanisamy, Renganathan Ramalakshmi, Muniappan Devibala, Maruthanayagam Saranya, Sekar Sivaranjini, R Thangavelu, Manickam Mahalingam

**Affiliations:** 1 Department of Integrative Oncology, Mirakle Integrated Health Centre, Pollachi, IND; 2 Department of Medical Oncology, Mirakle Integrated Health Centre, Pollachi, IND; 3 Department of Integrative Medicine, Mirakle Integrated Health Centre, Pollachi, IND; 4 Department of General Medicine, Mirakle Integrated Health Centre, Pollachi, IND

**Keywords:** brain tumor, high dose vitamin c, hyperthermia, infratentorial meningioma, oncothermia

## Abstract

Infratentorial meningiomas (IM) pose unique challenges in treatment due to their location and proximity to critical neurovascular structures such as cranial nerves, arteries, veins, etc. This case report presents the efficacy of an integrative medicine approach in managing IM, highlighting the multidisciplinary collaboration of modern medicine, yoga, naturopathy, and homeopathy. A 60-year-old male with IM, deemed unsuitable for surgery, underwent a tailored integrative medicine protocol involving oncothermia, ozone therapy, high-dose vitamin C, hydrogen inhalation, time-restricted feeding, hydrotherapy, biologicals, acupuncture, and yoga. Follow-up assessments revealed significant symptom reduction, stable biochemical markers, and tumor size reduction observed in MRI scans. While individual modalities have demonstrated benefits in cancer care, this case underscores the potential of integrating diverse therapies to address the multifaceted challenges of IM. The findings offer valuable insights into the evolving landscape of integrative medicine in brain tumor management, suggesting promise for improved patient outcomes and enhanced quality of life. Further research and clinical trials are warranted to comprehensively evaluate the safety and efficacy of integrative approaches in IM treatment.

## Introduction

Meningiomas are the most common intracranial tumors, making up more than a third of all primary central nervous system (CNS) tumors. Infratentorial meningiomas (IM) are a subtype located in the infratentorial compartment, below the tentorium cerebelli, and represent 7-12% of all meningiomas. Patients with IM often experience a range of symptoms, including ataxia, headaches, loss of fine motor skills, cognitive disturbances, and cranial nerve deficits. These symptoms vary depending on the tumor's location and size [1]. Surgery is considered the standard of care; however, IM presents unique challenges due to their proximity to critical neurovascular structures and the complexities involved in their surgical management [1,2]. There are brief reports on the use of integrative medicine approaches like oncothermia (modulated elector-hyperthermia) [3,4], high-dose vitamin C [5], hydrogen inhalation [6], intermittent fasting [7], yoga [8], and acupuncture [9] on the management of various tumors including the brain. To date, there is limited literature available regarding the application of complementary and alternative medicine (CAM) therapies in the treatment of IM. This case report contributes initial findings regarding the efficacy of an integrative medicine approach in managing IM.

## Case presentation

In January 2024, a 60-year-old male, a known case of IM, sought care at our integrative oncology facility, reporting numbness in both hands, diminished strength and grip, instability while walking, inability to lift the left leg, pronounced fatigue, and neck pain. Additionally, he expressed concerns about constipation and progressive weakness impeding his daily activities. The patient has a history of primary hypertension and dyslipidemia diagnosed since 2021.

Clinical findings

The patient's height measured 164 cm, weight recorded at 76.7 kg, with a body mass index (BMI) of 28.5 kg/m^2^. His pulse rate was 78 beats per minute, and blood pressure measured at 120/90 mmHg. He was taking Arbitel 40 (Telmisartan 40 mg) for hypertension and Rosave 10 (Rosuvastatin and Cholecalciferol) OD for dyslipidemia.

Diagnostic focus and assessments

**Figure 1:**
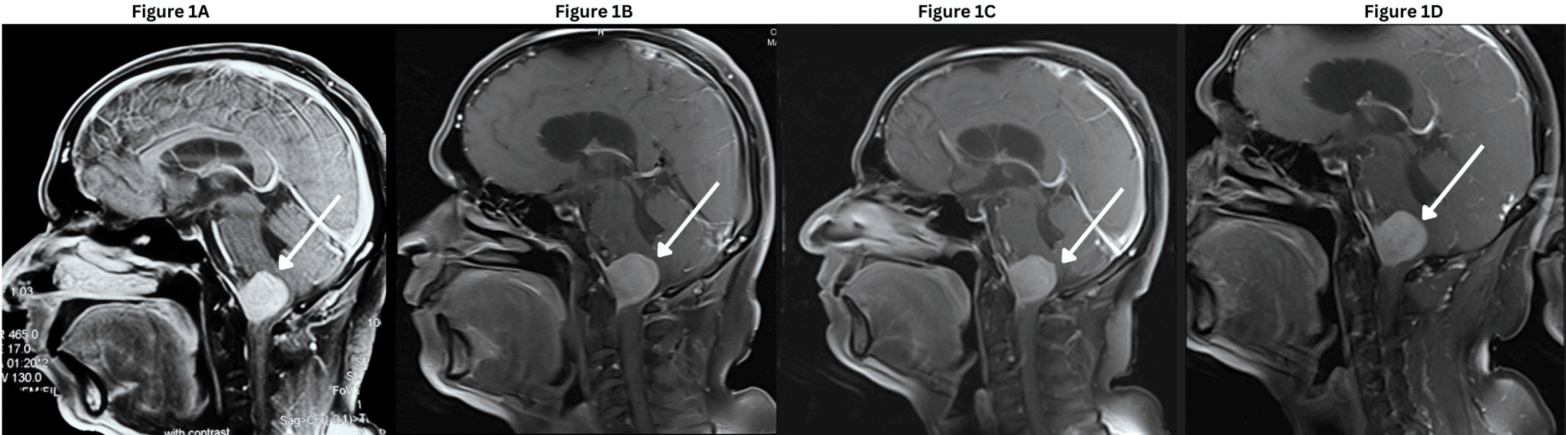
Changes in tumor size across time points Figure 1A: At diagnosis (June 2023) Figure 1B: On admission (January 2024) Figure 1C: After 24 sessions of oncothermia (April 2024) Figure 1D: After 36 sessions of oncothermia (July 2024)

He was diagnosed with IM following a magnetic resonance imaging (MRI) conducted in June 2023 after experiencing sudden functional disability. The MRI revealed an extra-axial mass lesion at the large anterior cervico-medullary junction, extending from the C1 vertebra, measuring 29x27x26 mm (APxTRxCC), compressing the medulla (Figure 1A). He was advised for a surgery, however, the surgery was termed risky by his oncosurgeon, which made the patient deciding against going for a surgery. He took homeopathic medications and underwent naturopathy treatments at various alternative medical settings for five months.

The second MRI on January 2024 revealed the progression of the disease, where the lesion increased in size measuring 3.1x3.2x2.8 cm (APxTRxCC), compressing and displacing medulla oblongata (Figure 1B). At baseline (January 2024), the blood work was done to collect complete blood count, renal function test, liver function test, glycosylated haemoglobin, immunoglobulin G, immunoglobulin E, free T3, free T4, thyroid stimulating hormone, lipid profile, C-reactive protein, D-Dimer, carcinogenic embryonic antigen (CEA) and lactate dehydrogenase.

This was followed up by an MRI of the brain after three months from baseline. Furthermore, we used the Edmonton Symptom Assessment Scale (ESAS) tool for measuring the changes in the symptoms [10], the European Organisation For Research And Treatment Of Cancer Quality of Life questionnaire (EORTC QLQ-C30) for measuring the quality of life [11], and the Chuang prognostic scale for predicting the survival [12]. The data is tabulated in Table 1. Further, we also assessed the glucose-6-phosphate dehydrogenase (G6PD) status of the patient to ensure the safety of administering intravenous vitamin C and ozone therapy.

**Table 1:** Changes in the biochemical, safety and psychological variables across the time RBC - Red blood cells (million/μL); Hb - Haemoglobin (g/dL); PCV - Packed cell volume (%); TC - Total leucocyte count (Cells/μL); N - Neutrophils (%); L - lymphocytes (%); E - Eosinophils (%); Platelets (Cells/μL); ESR - Erythrocyte sedimentation rate (mm/hr); FE - Ferritin (ng/mL); CEA - Carcinoembryonic antigen (ng/mL); LDH - Lactate dehydrogenase (U/L); D-Dimer (ng/mL); CRP - C-reactive protein (mg/dL); BMI - Body mass index; Total bilirubin (mg/dL); SGOT - Serum glutamic oxaloacetic transaminase (U/L); SGPT - Serum glutamic pyruvic transaminase (U/L); ALP - Alkaline phosphatase (U/L); Urea (mg/dl); Creatinine (mg/dl); eGFR - Estimated glomerular filtration rate (mL/min/1.73 m^2^); Uric acid (mg/dl); Na - Sodium (mEq/L); K - Potassium (mEq/L); IgE - Immunoglobulin E (IU/ml); IgG - Immunoglobulin G (mg/dl); Vitamin D - 25-hydroxy vitamin D (ng/mL); TC - Total cholesterol (mg/dl); TGL - Triglycerides (mg/dl); HDL - High density lipoprotein (mg/dl); LDL - Low density lipoprotein (mg/dl); VLDL - Very low density lipoprotein (mg/dl); SBP - Systolic blood pressure (mmHg); DBP - Diastolic blood pressure (mmHg); PR - Pulse rate (beats/minute); SPO2 - Saturation of peripheral oxygen; RR - Respiratory rate (breaths/minute); EORTC QLQ-C30 - European Organisation For Research And Treatment Of Cancer Quality of Life questionnaire

Changes in complete blood count, cancer markers and anthropometric profile																
Time points	Red blood cells	Hemoglobin	Packed cell volume	Total leucocyte	Neutrophils	Lymphocytes	Eosinophils	Platelets	Erythrocyte sedimentation rate	Ferritin	Carcinogenic embryonic antigen	Lactate dehydrogenase	D-DIMER	C-reactive protein	Weight	Body mass index kg/m^2^
January 2024	4.51	14.2	41.6	4700	52.1	38	3.9	238000	8	78.9	2.1	180	220	20	76.7	28.5
February 2024	4.74	14.7	46.5	5450	52.4	29.7	9.1	164000	7	NE	<1.7	174.4	146	12	74.2	27.6
April 2024	4.78	14.3	47.2	5460	30.9	18.2	3.5	235000	5	NE	<1.7	61.1	162	4	72.2	26.8
Safety and metabolic profiles across time points																
Time points	Liver Function						Glycosylated hemoglobin	Renal Function Test						Immunoglobulin E	Immunoglobulin G	Vitamin D
	Total Bilirubin	Serum Glutamic Oxaloacetic Transaminase	Serum Glutamic Pyruvic Transaminase	Alkaline Phosphatase	Total Protein	Albumin		Urea	Creatinine	Estimated Glomerular Filtration Rate	Uric acid	Sodium	Potassium			
January 2024	0.8	21.1	34.7	77	6.6	4.14	5.6	19.5	0.89	93	5.1	142	4.4	278	1100	12
February 2024	1.39	24	21	83	7	4.4	6.2	13	0.9	90	5.2	136	4.5	276.7	1080	25.6
April 2024	1.50	23	18	79	7.1	4.7	6.2	11	0.9	95	5.2	138	4.2	244	985	50.2
Time points	Cardiorespiratory profiles across time points										Chuang's Survival Score	European Organisation For Research And Treatment Of Cancer Quality of Life questionnaire				
	Total cholesterol	Triglycerides	High-density lipoprotein	Low-density lipoprotein	Very low-density lipoprotein	Systolic blood pressure	Diastolic blood pressure	Pulse rate	Saturation of peripheral oxygen	Respiratory rate		Global	Functional	Symptoms		
January 2024	147	105.7	43.68	82.6	21.14	120	90	78	98%	22	3	50	64.4	92.3		
February 2024	127	67	47	67	13	120	70	90	97%	22	NE	NE	NE	NE		
April 2024	131	92	47	66	18	110	90	74	99%	24	0.7	50	55.5	20.5		
	Edmonton Symptom Assessment Scale															
Time points	Nausea	Depression	Anxiety	Drowsy	Appetite	Wellbeing	Breathlessness	Pain	Tiredness							
January 2024	0	3	1	7	1	6	7	6	7							
April 2024	0	0	0	0	0	0	0	0	5							

Methods

After conducting an extensive case review and obtaining informed consent, a collaborative team of physicians specializing in modern medicine, yoga and naturopathy, and homeopathy devised an integrative medicine protocol. The treatment regimen was tailored and administered on an outpatient basis.

Therapeutic focus and assessment

The therapeutic protocol included oncothermia, ozone therapy, intravenous high-dose vitamin C, hydrogen inhalation, time-restricted feeding, hydrotherapy, pulsed-electromagnetic field, acupuncture, hydrosun, biologicals and yoga. He was also provided with nutritional supplements to address his functional and nutritional deficiencies. The detailed treatment protocol is tabulated in Table 2.

**Table 2:** Integrative medicine protocol used

Intervention	Frequency of intervention
Counselling	Daily
Oncothermia (Oncotherm HY300, Budapest, Hungary)	Alternate days (A total of 36 sessions was provided with a cooling period of 3 weeks in-between each 12 sessions)
High-dose vitamin C therapy	2-3 times per week, the total dose not exceeding 90 grams per week.
Ethylenediaminetetraacetic acid (EDTA) Chelation	EDTA chelation was performed once in 10 days for removing the heavy metal toxins.
Ozone therapy	Daily (Ozone is administered in the form of rectal insufflation/Ear insufflation/ saline ozone, Minor autohaemotherapy and major autohaemotherapy).
Hydrogen inhalation	Daily inhalation of hydrogen for 30 minutes using a hydrogen generator (AT_HO-600 Hydrogen Inhalation Machine, Athena, Mumbai, India)
Diet therapy	Daily (A complete Millet-based, sugar-free, maida-free, refined grains-free diet was advised to our patients. This also includes carrot, beetroot, apple juice, fresh vegetable salads, and soups.)
Liposomal curcumin therapy	Daily (5-10 ml liposomal curcumin), Mirakle, Coimbatore, India
Pulsed electromagnetic field therapy	Daily for 30 minutes, exposed to PEMF using a controlled magnetic field assembly (PULSATRON at 10 Hz field frequency), Madras Institute of Magnetobiology (MIM), Chennai.
Probiotic supplementation	Daily (Two doses of Liv-Bio alpha Pre and probiotic supplements), Liv Bio Pharma, Poovarany, India
Co-Q enzyme therapy	Daily (5-15 ml liposomal Co-Q 10 enzyme), Mirakle, Coimbatore, India
Coffee enema	Once in a week
Hydrosun	Daily (Hydrosun light therapy) using Hydrosun 750, Hydrosun Medizintechnik, Müllheim, Germany
Acupuncture	Scalp acupuncture- Motor Control area, Other acupuncture points like large intestine 4, stomach 36, spleen 6, Back-Shu points and Ah-shi points were punctured daily for 30 minutes. One week gap was given between each 10 sittings.
Yoga therapy	Daily 30-45 minutes (Includes pranayama, meditation and asanas)
Enema	Once in a week to relieve constipation
Cold abdominal pack	Daily tied for 45 minutes
Hot foot bath	As and when there is fatigue and headache
High-dose vitamin D supplementation	Once in a week (60000 IU after monitoring the levels of parathyroid hormones and ionized calcium levels.)

Follow-up

**Figure 2:**
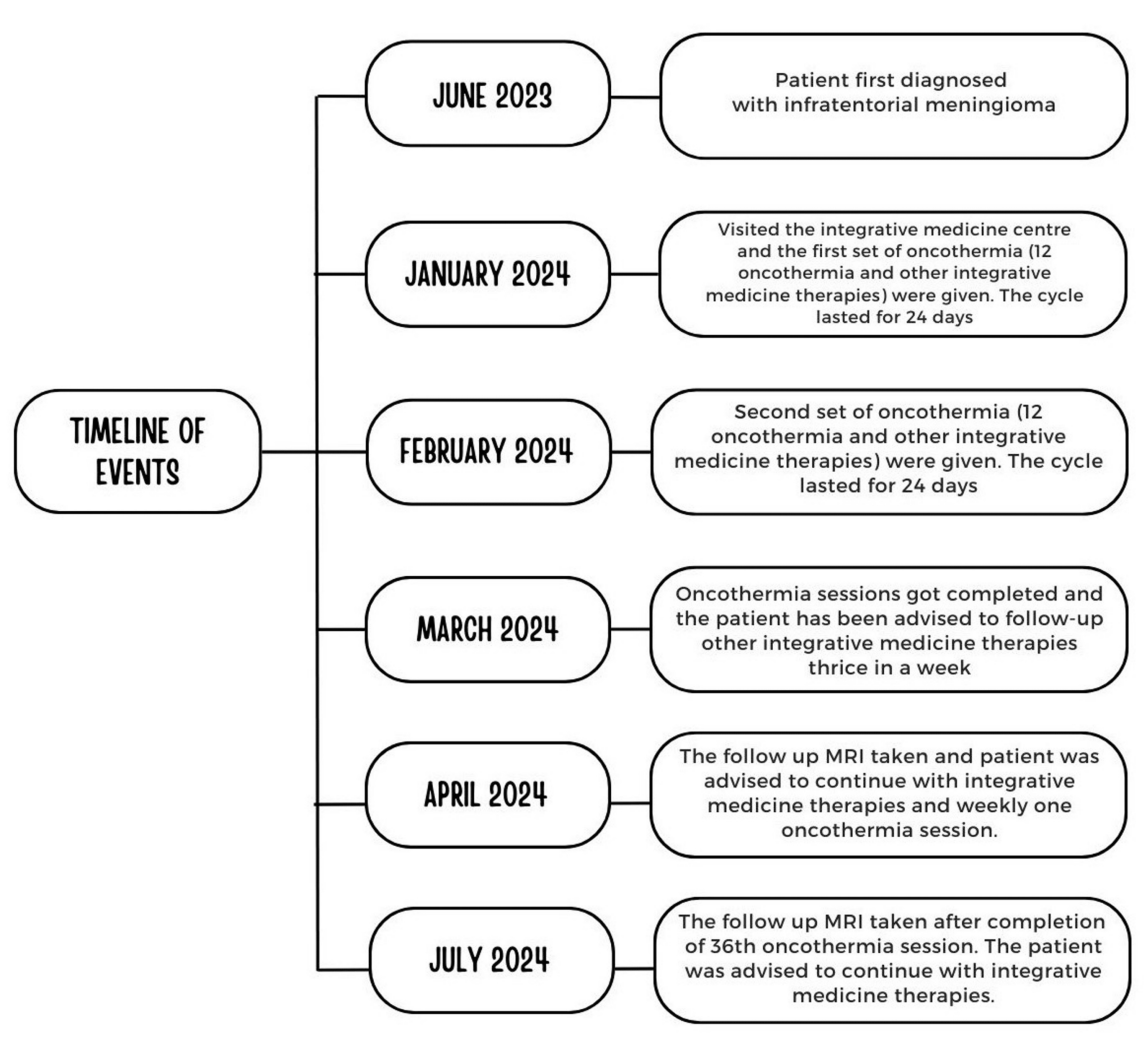
Timeline of events

The patient underwent 36 sessions of oncothermia over a span of 90 days, complemented by various integrative medicine modalities over a period of five months, as outlined in Table 2. Additionally, a time-restricted diet and other nutritional supplements (Table 2) were recommended during the follow-up period. Weekly follow-ups were advised and are currently ongoing. A comprehensive timeline of events is depicted in Figure 2.

Outcomes

The patient has exhibited a significant reduction in symptoms, and their biochemical markers remained stable throughout both the treatment period and the subsequent follow-up. Furthermore, a decrease in the volume of the tumor lesion was observed (2.7x3.1x2.7cm) compared to the MRI taken on January 2024 (3.1x3.2x2.8 cm) signifying a positive impact of the treatment. Figure 1C illustrates the changes in the brain MRI. The patient reported improvements in postural balance, gripping power, and memory following the therapies. We observed a significant improvement in the mental health, physical health and symptoms measured by the EORTC QLQ-C30 and ESAS. We repeated an MRI after one week of completion of the 36th session of oncothermia, which suggested no interval change in the size of the tumor lesion (2.8x3.5x2.8 cm) compared to the previous MRI on April 2024 (Figure 1D).

## Discussion

IM stands out as one of the most formidable brain tumors, posing significant challenges in terms of morbidity and mortality. This case report offers an initial investigation into the efficacy of an integrative medicine protocol for managing IM. Given the limitations of conventional approaches to IM treatment, the integrative medicine protocol presented herein shows promise.

This protocol primarily utilizes oncothermia (EHY 2030, Oncotherm Kft, Budaörs, Hungary) as a targeted cancer therapy. Oncothermia, a form of modulated electrohyperthermia, uniquely targets cancer cells selectively, setting it apart from other conventional hyperthermia methods [13]. This technology employs microheating to induce immune-stimulating effects, including apoptosis, leukocyte activation, and ultimately, cell death [14]. Reports suggest the usefulness of oncothermia in various types of cancers such as stomach, colon, rectum, lung, liver, pancreas, bladder, ovary and kidneys [15]. However, this is the first case report to show the efficacy of oncothermia as an integrated treatment option for IM.

High-dose vitamin C constituted another significant therapeutic component in this protocol. Its usage is increasingly gaining traction as an anti-cancer therapy [16]. At high doses, vitamin C enhances the production of reactive oxygen species (ROS) within cancer cells, increasing oxidative stress and leading to their destruction. Additionally, it is suggested that high-dose vitamin C promotes the influx of iron into cancer cells while inhibiting its efflux, thereby inducing cell death through ferroptosis [16,17]. Earlier reports also suggest the usefulness of high-dose vitamin C infusion as a supportive therapy in improving the quality of life and survival time in patients with Glioblastoma Multiforme [18]. This report contributes to the evidence-based literature on the use of high-dose vitamin C in cancer, particularly in cases involving the brain.

Ozone therapy has been administered in various forms, including rectal and ear insufflation, as well as minor and major autohemotherapies. Evidence suggests its efficacy in the treatment of brain tumors [19,20]. The anti-carcinogenic effect of ozone therapy is proposed to stem from its role in modulating the production of reactive oxygen species (ROS) and alleviating the hypoxic state of cancer cells [19]. The findings from the present study also support the positive integration of ozone therapy in the management of brain tumors.

In addition, hydrogen peroxide inhalation was also a crucial component of this integrative medicine protocol. A recent animal study has demonstrated that hydrogen inhalation can reduce the size, invasiveness, and colony-forming ability of glioma cells [6]. Similarly, numerous clinical trials and case studies have been published supporting the antitumor effects of hydrogen inhalation [21]. In a recent case report, Chen et al. demonstrated a significant reduction in brain tumor size and an enhanced survival time following hydrogen inhalation as a monotherapy [22]. While our data did not involve hydrogen inhalation as a monotherapy, it does suggest the potential role of hydrogen inhalation as part of an integrative therapy approach.

Diet plays a major role in the management of every chronic disease, including cancer. Our patient followed a strict millet-based diet within a restricted time window (14:10 hours), during which he consumed food ad libitum for 10 hours and adhered to water fasting for 14 hours. Refined sugar, white rice, maida, and dairy products were completely excluded from the diet. Millets are rich in micronutrients and exhibit superior antioxidant properties compared to refined grains. Furthermore, they enhance immune surveillance, reduce fatigue, and halt carcinogenesis [23]. Moreover, research has shown that Time-Restricted Feeding (TRF) can impact essential pathways related to cancer progression, such as insulin signaling, inflammation, cellular metabolism, and the enhancement of autophagy [24,25].

Hydrotherapy procedures like enema, hot foot bath and abdominal pack were included in this protocol. A cold water enema was administered to relieve constipation, while an abdominal pack was used to enhance cardiometabolic functions. The abdominal pack involves soaking a cotton cloth in cold water, wringing it out, and applying it to the abdomen. A dry cloth and flannel are then layered over the moist cloth to minimize evaporation. This technique primarily functions through thermoregulation [26]. Hot foot bath helps in relieving fatigue and improve sleep among cancer patients [27].

Pulsed electromagnetic field (PEMF) application was administered daily for 30 minutes in this case. PEMF has been proposed to induce cell death and senescence in cancer cells by reducing proliferation rates and inhibiting neovascularization [28,29]. However, to date, no studies have investigated whether PEMF can have a similar impact on brain tumors. Given the multidisciplinary integrative approach employed in this protocol, it is not possible to attribute the observed changes in the present case solely to PEMF. Thirty sittings of acupuncture were provided for this patient to improve his musculoskeletal weakness, gait irregularities and gripping. Earlier studies reported the usefulness of acupuncture in improving the gait and functional ability in neurological disturbances [30,31].

Hydrosun, a form of water-filtered infrared-A irradiation with wavelengths ranging between 780-1400 nm, presents a novel approach with significant implications for medical practice. Primarily utilized in wound healing, its potential extends to inducing tissue regeneration and repair [32]. Moreover, it has been explored in conjunction with chemotherapy and radiation therapy for cancer treatment [33]. However, it is imperative to note that further research and clinical trials are necessary to comprehensively understand Hydrosun's efficacy, safety, and optimal applications in medical settings. Mental health deficiencies like anxiety, stress and depression are very common among cancer patients. Our patient underwent yoga therapy every day to cope up with the stress associated with his underlying condition. Earlier studies also have recommended yoga as a promising intervention to handle stress and improve health in patients with brain cancers [8,34]. Presently, the patient is visiting our clinic and undergoing the follow-up treatments like high-dose vitamin C, chelation, ozone therapies, acupuncture, yoga and weekly one session of oncothermia.

Although the results of this study are promising, it is important to note that as a case report, it cannot be regarded as conclusive evidence. The findings are limited to a single case, and therefore, generalizability is restricted. Further research involving larger sample sizes and longer follow-up periods is necessary to better understand the potential of the integrative oncology approach in achieving complete remission. Despite the inherent limitations of a case report, this study highlights the potential of integrative oncology in the complex management of infratentorial meningioma. It provides valuable insights and opens avenues for further therapy and research. The innovative combination of oncothermia, lifestyle modification and complementary therapies showcased in this report offers a promising direction for future investigations and clinical applications.

## Conclusions

In the realm of formidable brain tumors like infratentorial meningioma (IM), where conventional treatment options face limitations, this case report explores the efficacy of an integrative medicine protocol. The patient expressed his happiness with the promising prognosis he received, both in terms of reduction in tumor size and improvement in quality of life. The protocol, encompassing various modalities, demonstrates promise in managing IM. Being a multi-faceted approach, we cannot identify which therapy is superior to the other, which needs to be explored in future studies. While these findings suggest promise, the study underscores the need for further research and clinical trials to ascertain the safety and efficacy of these integrative approaches comprehensively. Nonetheless, this report adds valuable insights to the evolving landscape of integrative medicine in brain tumor management, offering hope for improved patient outcomes and enhanced quality of life.
